# Trends in Emergency Department Visits Among Undocumented Patients

**DOI:** 10.1001/jamanetworkopen.2026.14499

**Published:** 2026-05-26

**Authors:** Rama A. Salhi, Sarah Rapaport, Melissa A. Meeker, Fiona Danaher, Matthew Gartland, Margaret E. Samuels-Kalow

**Affiliations:** 1 Department of Emergency Medicine, Massachusetts General Hospital, Boston; 2 Department of Pediatrics, Massachusetts General Hospital, Boston; 3 Department of Medicine, Massachusetts General Hospital, Boston

## Abstract

This cohort study examines the association between recent immigration enforcement policy changes with emergency department visits by undocumented patients, with analysis by insurance status and preferred language as proxies for documentation status.

## Introduction

Recent federal immigration policy changes have greatly influenced the landscape of health care delivery in the US.^1,2^ In particular, on January 20, 2025, the Department of Homeland Security issued a memorandum rescinding the Protected Areas Policy.^3^ As identification of undocumented patients in administrative records is appropriately limited, proxies, such as language, are often used to extrapolate impacts on care-seeking among immigrants.^4,5^ With ongoing changes affecting immigrant patients and given the limited ability to track the impact on care-seeking, we sought to (1) use an emergency insurance product available to undocumented patients in Massachusetts (MassHealth Limited) to evaluate the association of recent policy changes with emergency department (ED) visits and (2) evaluate the extent to which analysis by preferred language may or may not demonstrate the same utilization patterns.

## Methods

This cohort study included all patients presenting to an ED of 5 hospitals in a large, regional health care system from August 6, 2024, to July 7, 2025. This period spanned the 24 weeks before (preperiod) and on/after January 21, 2025 (postperiod). The study conforms to the STROBE guideline and was deemed exempt by the Mass General Brigham IRB with a waiver of informed consent due to large sample sizes and minimal risk to participants.

Because documentation status is appropriately not collected in the health record, 2 proxy methods for undocumented status were used: a combined MassHealth Limited or missing insurance (MHL/missing) variable and language. Descriptive analyses assessed ED visits in the preperiod and postperiods by insurance type, overall, and stratified by preferred language, age (<18 or ≥18 years), and high acuity (defined as emergency severity index [ESI] level 1) (eMethods in Supplement 1). Encounter rates (percentage of total weekly encounters) by insurance type with smoothed trends were generated by locally estimated scatterplot smoothing. We conducted all analyses in R, version 4.3.1 (R Foundation).

## Results

Of 357 643 visits included in the study period (preperiod n = 178 697; postperiod n = 178 946), 87.8% were adults, and 10.3% had MHL/missing insurance (Table). Among adult patients with MHL/missing insurance, there were 16 666 visits in the preperiod and 14 799 visits in the postperiod, a decrease of 11.2% (95% CI, 10.7%-11.7%). Among pediatric patients with MHL/missing insurance, there were 2888 visits in the preperiod and 2500 visits in the postperiod, a decrease of 13.4% (95% CI, 12.2%-14.7%). Looking at visits with ESI 1, estimates for patients with MHL/missing insurance were higher in the postperiod, though CIs overlapped (preperiod, 6.9% [95% CI, 4.7%-9.3%] vs postperiod, 7.5% [95% CI, 5.2%-9.9%]).

**Table.** Frequency and Percentage of ED Encounters at 24 Weeks Before and After January 21, 2025, by Insurance Type and Language

Population	No. (%) [95% CI]	
	Before (n = 178 697)	After (n = 178 946)
**Overall**		
Insurance		
MHL or missing	19 554 (10.9) [10.7-11.2]	17 299 (9.7) [9.4-9.9]
Medicare or Medicaid	98 754 (55.3) [55.0-55.5]	100 427 (56.1) [55.9-56.4]
Commercial	55 733 (31.2) [30.9-31.4]	56 692 (31.7) [31.4-31.9]
Other^a^	4656 (2.6) [2.4-2.8]	4528 (2.5) [2.3-2.8]
Language		
English	146 786 (82.1) [82.0-82.3]	148 056 (82.7) [82.6-82.9]
Spanish	20 792 (11.6) [11.5-11.8]	20 439 (11.4) [11.3-11.6]
Portuguese	2192 (1.2) [1.1-1.4]	1816 (1.0) [0.8-1.2]
Haitian Creole	2086 (1.2) [ 1.0-1.3]	2000 (1.1) [0.9-1.3]
Other^b^	5567 (3.1) [2.9-3.3]	5622 (3.1) [3.0-3.3]
Declined, unavailable, or missing	1274 (0.7) [ 0.5-0.9]	1013 (0.6) [0.4-0.7]
**Pediatric**		
Insurance		
MHL or missing	2888 (13.1) [12.4-13.8]	2500 (11.6) [10.9-12.4]
Medicare or Medicaid	9769 (44.2) [43.5-44.9]	9736 (45.3) [44.6-46.1]
Commercial	9258 (41.9) [41.2-42.6]	9011 (42.0) [41.2-42.7]
Other^a^	190 (0.9) [0.1-1.6]	231 (1.1) [0.4-1.8]
Language		
English	16 222 (73.4) [72.8-74.0]	15 822 (73.7) [73.1-74.3]
Spanish	4215 (19.1) [18.5-19.7]	4236 (19.7) [19.1-20.3]
Portuguese	653 (3.0) [2.4-3.5]	522 (2.4) [1.8-3.0]
Haitian Creole	357 (1.6) [1.0-2.2]	320 (1.5) [0.9-2.1]
Other^b^	461 (2.1) [1.5-2.7]	451 (2.1) [1.5-2.7]
Declined, unavailable, or missing	197 (0.9) [0.3-1.5]	127 (0.6) [0.0-1.2]
**Adult**		
Insurance		
MHL or missing	16 666 (10.6) [10.4-10.9]	14 799 (9.4) [9.1-9.7]
Medicare or Medicaid	88 985 (56.8) [56.6-57.1]	90 691 (57.6) [57.3-57.9]
Commercial	46 475 (29.7) [29.4-29.9]	47 681 (30.3) [30.0-30.5]
Other^a^	4466 (2.9) [2.6-3.1]	4297 (2.7) [2.5-3.0]
Language		
English	130 564 (83.4) [83.2-83.6]	132 234 (84.0) [83.8-84.2]
Spanish	16 577 (10.6) [10.4-10.8]	16 203 (10.3) [10.1-10.5]
Portuguese	1539 (1.0) [0.8-1.2]	1294 (0.8) [0.6-1.0]
Haitian Creole	1729 (1.1) [0.9-1.3]	1680 (1.1) [0.9-1.2]
Other^b^	5106 (3.3) [3.1-3.4]	5171 (3.3) [3.1-3.5]
Declined, unavailable, or missing	1077 (0.7) [0.5-0.9]	886 (0.6) [0.4-0.7]
**ESI level 1**		
Insurance		
MHL or missing	104 (6.9) [4.7-9.3]	122 (7.5) [5.2-9.9]
Medicare or Medicaid	1042 (69.5) [67.2-71.9]	1080 (66.5) [64.2-68.8]
Commercial	314 (20.9) [18.7-23.3]	387 (23.8) [21.5-26.2]
Other^a^	40 (2.7) [0.4-5.1]	36 (2.2) [0.0-4.6]
Language		
English	1242 (82.8) [81.0-84.6]	1353 (83.3) [81.6-85.0]
Spanish	145 (9.7) [7.9-11.5]	132 (8.1) [6.5-9.9]
Portuguese	12 (0.8) [0.0-2.6]	12 (0.7) [0.0-2.5]
Haitian Creole	28 (1.9) [0.1-3.7]	32 (2.0) [0.3-3.7]
Other^b^	60 (4.0) [2.2-5.8]	77 (4.7) [3.1-6.5]
Declined, unavailable, or missing	13 (0.9) [0.0-2.7]	19 (1.2) [0.0-2.9]

Abbreviations: ED, emergency department; ESI, Emergency Severity Index; MHL, MassHealth Limited.

^a^ Includes automobile, workers' compensation, carceral health, international, military, government employee, hospice, Veterans’ Affairs, and charitable insurance sources.

^b^ Includes more than 100 other languages.

When examining language, patients with Portuguese as a preferred language had the highest rates of MHL/missing insurance (55.2%; 95% CI, 53.6%-56.8%) as compared to English (6.3%; 95% CI, 6.1%-6.5%), Spanish (30.5%; 95% CI, 30.0%-31.0%), Haitian Creole (18.6%; 95% CI, 17.2%-20.1%), or other (13.5%; 95% CI, 12.6%-14.4%). Among nonmissing languages, patients with Portuguese as a preferred language showed the most pronounced overall drop in visits (decline of 17.2%; 95% CI, 15.6%-18.8%) (Table, Figure).

**Figure.**
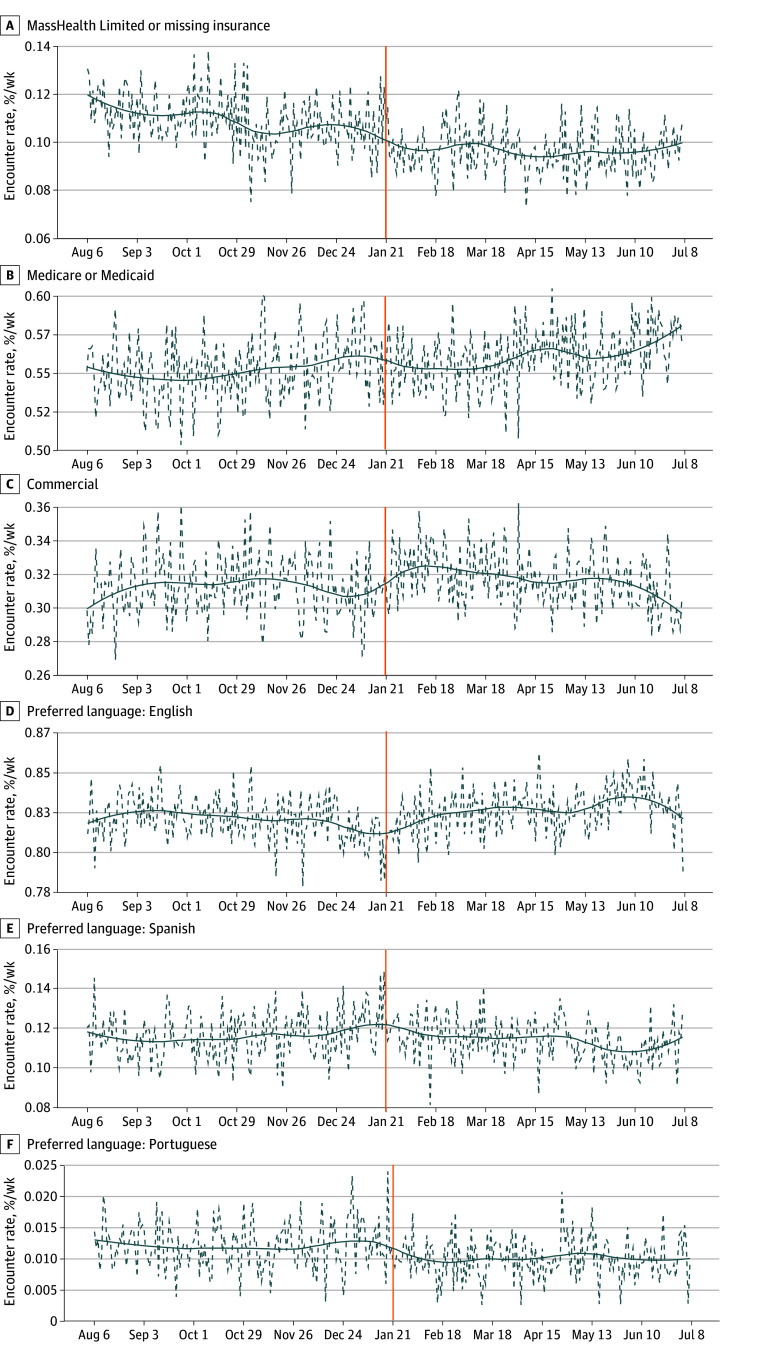
Line Graphs of Emergency Department (ED) Encounter Rates ED encounter rates by insurance type (A-C) and preferred language (D-F) in the 24 weeks before and after January 21, 2025 (indicated by vertical orange line). Dashed lines represent weekly encounter rates; solid lines are the smoothed trends generated by locally estimated scatterplot smoothing.

## Discussion

In this cohort study, we observed 2 trends. First, we observed a decline in ED visits for patients with MHL/missing insurance following the rescission of the Protected Areas Policy. This may, in part, reflect national policy changes leading to social climate change in the setting of immigration enforcement efforts, including some occurring at medical facilities. Of note, Boston and many surrounding cities have sanctuary policies that may afford some protection against health care avoidance. Communities without sanctuary policies might demonstrate more dramatic patterns of health care avoidance. Second, we found that use of preferred language did not consistently identify the decline seen when using insurance, suggesting language may be an inaccurate proxy. Notably, the observed trends among patients with Portuguese as a preferred language may be related to local migration patterns from Brazil.

Limitations of our study include the state-specific nature of Medicaid insurance products, which limits the generalizability of our approach to states with high uninsurance rates or without emergency products. While MHL is the only insurance product that undocumented patients are eligible for in Massachusetts, other individuals (such as those seeking asylum or those with permanent resident cards who have been in the US for <5 years) also use the product. Thus, results specific to undocumented patients may be attenuated. Additionally, while we evaluated temporal associations, we cannot establish a causative relationship, although prior work suggests that there is likely a relationship between policy and care-seeking trends.^6^ Finally, though this trend could not be evaluated in our study, a proportion of the decline in ED visits may be secondary to patients leaving the community (eg, through voluntary or involuntary relocation). Future research and policy focused on upholding medicine’s ethical obligation to care delivery must use caution in drawing false or attenuated conclusions from the available data while also balancing policy-relevant data with the obligation to avoid collecting information that may cause harm.

## Notes

Supplement 1.
eMethods.

Supplement 2.
Data Sharing Statement
