# Supplementary material for: Trends in Emergency Department Visits Among Undocumented Patients
Source: JAMA Netw Open. 2026 May 26;9(5):e2614499. doi: 10.1001/jamanetworkopen.2026.14499 (PMC13213525; doi:10.1001/jamanetworkopen.2026.14499)
Supplement: Supplement 1. — eMethods. [file jamanetwopen-e2614499-s001.pdf]

Supplemental Online Content

Salhi RA, Rapaport S, Meeker MA, Danaher F, Gartland M, Samuels-Kalow ME. Trends in emergency department visits among undocumented patients. *JAMA Netw Open*. 2026;9(5):e2614499. doi:10.1001/jamanetworkopen.2026.14499

eMethods

This supplemental material has been provided by the authors to give readers additional information about their work.

© 2026 Salhi RA et al. JAMA Network Open.

eMethods

As detailed in the main text, two proxy methods were utilized. First, we leveraged the Massachusetts health insurance landscape, which has policies in place to cover all residents, including those who are undocumented, making the rate of uninsured people approximately 1.7%.[9] As such, a combined MassHealth Limited or missing insurance variable was utilized as a proxy for documentation status. MassHealth Limited is a state-administered Medicaid program in Massachusetts that covers emergency medical care and does not require citizenship documentation. Once documentation status is obtained, health insurance is typically transitioned to more comprehensive coverage. Other insurance categories examined were public insurance other than MassHealth Limited (all other Medicare and Medicaid products) and commercial insurance. Second, we analyzed preferred language, a common approach to approximating immigrant populations, to trend ED visits.[1–3] A descriptive analysis explored changes in ED visits in the pre- and post-periods by insurance type, presented overall and stratified by: preferred language, age (<18, ≥18), and acuity. Acuity was defined by the assigned emergency severity index (ESI), a commonly used triage scoring system where 1 is the highest acuity (e.g., cardiac arrest or septic shock). Confidence intervals for the proportions by insurance group, presented overall and within sub-cohorts, were calculated using simultaneous estimation of multinomial proportions. No tests of association or hypothesis testing were generated as part of this analysis.

© 2026 Salhi RA et al. JAMA Network Open.
