# Supplementary material for: Trends in Emergency Department Visits Among Undocumented Patients
Source: JAMA Netw Open. 2026 May 26;9(5):e2614499. doi: 10.1001/jamanetworkopen.2026.14499 (PMC13213525; doi:10.1001/jamanetworkopen.2026.14499)
Supplement: Supplement 2. — Data Sharing Statement [file jamanetwopen-e2614499-s002.pdf]

Data Sharing Statement

Salhi. Trends in Emergency Department Visits Among Undocumented Patients. *JAMA Netw Open*. Published May 26, 2026. doi:10.1001/jamanetworkopen.2026.14499

Data
Data available: No

Additional Information
Explanation for why data not available: EHR data was utilized for the presented analysis and cannot be shared due to institutional guidelines. The data dictionary can be provided upon request.
